# Challenges and opportunities of sensory plasticity after SCI

**DOI:** 10.3389/fphys.2013.00231

**Published:** 2013-08-27

**Authors:** Jeffrey C. Petruska, Charles H. Hubscher, Alexander G. Rabchevsky

**Affiliations:** ^1^Department of Anatomical Sciences and Neurobiology, Kentucky Spinal Cord Injury Research Center, University of LouisvilleLouisville, KY, USA; ^2^Department of Physiology, Spinal Cord and Brain Injury Research Center, University of KentuckyLexington, KY, USA

**Keywords:** spinal cord injury (SCI), sensory plasticity, sensory perceptions, sensory neurons, sensory systems

Even in cases of spinal cord injury (SCI) where sensory perceptions do not arise from stimuli applied to below-level regions, sensory input to the spinal cord, carried by spinal sensory afferents, still occurs and influences the central and autonomic nervous systems (CNS, ANS). This is true also of the vagal system which provides non-spinal innervation of viscera below many spinal cord injuries. It is therefore important to consider (1) how the neurochemical, anatomical, and electrophysiological properties of these sensory neurons, and the processing of the inputs by the CNS and ANS, is altered by SCI, (2) whether and how they may play a role in pathologies, and (3) how they may interact with treatment strategies. This Research Topic addresses plasticity of sensory systems after SCI, with a non-exclusive focus on systems below the level of the injury.

## Post-SCI autonomic dysfunctions

The ANS controls systems below the level of consciousness and this is often taken for granted until something goes awry. Those living with SCI are acutely aware of the functions regulated, or more often dysregulated, by the ANS. One of the most pressing of these issues is autonomic dysreflexia (AD), a chronic and common hypertensive syndrome essentially unique to the high-level SCI community. It rarely arises acutely after injury (Krassioukov et al., 2003; Krassioukov, 2004), suggesting mechanisms beyond just loss of spinal sympathetic outflow regulation by the brain, and experimental evidence suggests that various forms of plasticity in numerous cell-types may contribute (e.g., Taylor and Schramm, 1987; Chau et al., 2000; Teasell et al., 2000; Rabchevsky, 2006; Schramm, 2006; Brown and Weaver, 2012). AD is generally considered an episodic pathology with bouts initiated and maintained by a physiological trigger, and is treated symptomatically and by finding and removing the stimulus. Continuing refinements in our understanding and measurements suggest that the severe clinical bouts that garner the most attention may be the tip of the iceberg of a much more insipid and persistent condition (e.g., Claydon et al., 2006; Krassioukov and Claydon, 2006). The most frequent triggers of AD appear to be noxious stimuli below the injury level [anything from a full bladder, an impacted bowel or a pressure ulcer to an ingrown toenail or simply having new shoes tied too tightly (e.g., Krassioukov et al., 2009)], placing focus onto plasticity in nociceptive sensory neurons (Ramer et al., 2012) for identifying potential mechanisms and treatments (Rabchevsky et al., 2012), though fundamental questions remain regarding the actual trigger in humans and experimental model systems (Macefield et al., 2012).

Additional autonomic functions are served and mediated by the vagal system, which is not directly impacted by experimental SCI and most clinical SCI. This vital and widespread system is nonetheless affected by SCI in terms of changes to electrical and chemical properties of neurons and changes in their connectivity (Kaddumi and Hubscher, 2007a,b; Holmes, 2012).

## Pain mechanisms and treatment

Chronic pain is not a consequence of SCI that is obvious to the casual observer, yet it is one of the most common post-SCI conditions and most impactful on the quality of life of SCI individuals (e.g., Finnerup and Baastrup, 2012). There are numerous mechanisms by which SCI-related pain can arise, some of which we are only now identifying, yet these are still poorly understood and there are few reliable treatments (e.g., Felix et al., 2007; Cruz-Almeida et al., 2009). The effect of SCI on primary sensory neurons is an emerging topic of investigation (Huang et al., 2006; Shortland et al., 2006; Ramer et al., 2012; Walters, 2012) as a possible mechanism of SCI-related pain and other pathologies such as AD (e.g., Widerstrom-Noga et al., 2004). New approaches to applying knowledge of nociceptive mechanisms are also being tested as potential treatments for SCI-related sensory pathologies (Gupta and Hubscher, 2012; Lee et al., 2012; Rabchevsky et al., 2012; Ramer et al., 2012).

## Effects of post-SCI training

In addition to a variety of forms of maladaptive plasticity, the spinal cord caudal to an injury which largely or completely separates it from the brain is capable of considerable and lasting adaptive plasticity, particularly activity-dependent plasticity (e.g., Edgerton et al., 1992; Hodgson et al., 1994; De Leon et al., 1999; Edgerton et al., 2001; Frigon and Rossignol, 2006), with some of this plasticity involving the sensory neurons (e.g., De Leon et al., 2001; Petruska et al., 2007). The spinal cord is capable of interpreting afferent input to learn a task and to counter perturbing forces or avoid obstacles placed in the path of hindlimbs stepping on a treadmill, and even retaining this information for a short time without reinforcement (Zhong et al., 2012). This collective work suggests that the spinal cord is capable of learning (see also Ferguson et al., 2012a,b; Grau et al., 2012), and may be capable of processes akin to formation of short- and long-term memory.

Generally the effects of training appear to be task-specific. For example, when an SCI animal is trained to step on a treadmill, this behavior improves, but the performance of other tasks, such as standing, does not improve (Edgerton et al., 1997; De Leon et al., 1998, 1999). However, training does appear to have effects on some processes outside of the trained task. In animal models there are demonstrations of reduced spasticity (Bose et al., 2012), and reduced nociception (Wolpaw and Tennissen, 2001; Hutchinson et al., 2004; Martin Ginis and Latimer, 2007; Herrity et al., 2012).

More recently, principles identified from animal experiments have been applied to human experiments and clinical treatment with some success (Behrman et al., 2005; Barbeau et al., 2006; Dobkin et al., 2006; Harkema, 2008; Edgerton and Roy, 2009; Harkema et al., 2011). However, the field still has much to discover in terms of the characteristics of spinal plasticity, the necessary and sufficient influencing factors, as well as certain measures of systems, molecular, and cellular mechanisms that enable, facilitate, and inhibit such adaptive plasticity.

## Mechanisms regulating spinal learning

Research on post-SCI training focuses on optimizing functional recovery and identifying relevant principles from the sensorimotor integration perspective. Another approach has examined the principles of learning that may be at play in the spinal cord (Ferguson et al., 2012a,b; Grau et al., 2012), with important concepts emerging about extrinsic factors *interfering* with successful spinal learning (i.e., training). Given the relative success of activity-based therapies in both animal and human experiments and the significant effort and resources dedicated to optimizing these approaches for clinical gain, we must also identify factors that inhibit recovery (e.g., Caudle et al., 2011; Ferguson et al., 2012a,b).

In this context it is intriguing that many clinical trials have exclusion criteria related to conditions that would be painful for spinal-intact individuals (bladder infection, pressure ulcer, tissue damage, etc.). Common among front-line therapists are anecdotes of discovering skin abrasions, treadmill harnesses pinching skin, bladder infections, and other covert noxious conditions in patients whose training sessions were unexpectedly going poorly. These anecdotes suggest that the powerful influence of the spinal nociceptive system on the spinal motor system known from animal work is also at play in SCI patients/subjects. Unfortunately, these accounts are not regularly included in data collection, limiting assessments of the role of nociception in activity-dependent therapies.

These concepts may be involved in other spinal processes. For example, systems that are accustomed to a certain level and pattern of activity can “fall out of tune” (e.g., Lundbye-Jensen and Nielsen, 2008). Also, growth of nociceptive afferent terminals within the cord contributes to AD (e.g., Cameron et al., 2006; Brown and Weaver, 2012). However, repetitive natural stimulation, determined to be accompanied by intraspinal sprouting of afferents, reduces nociceptive reflexes (Conde and Komisaruk, 2012). Collectively this suggests that the functional outcome of intraspinal afferent growth may be dependent on the pattern of information carried by those afferents and the context of the intraspinal growth. Perhaps intraspinal growth that is uncoupled from specific patterned input becomes maladaptive, while growth associated with patterned input is associated with adaptive outcomes [Conde and Komisaruk, 2012; Ferguson et al., 2012a,b; Grau et al., 2012; and discussed in Petruska et al. (2007) and Maier et al. (2009)].

## Effects of SCI on neural tissue remote from the injury

Considering points of similarity and difference among experimental observations makes it clear that many characteristics of the injury model can have significant impact on the outcomes being measured (e.g., Cote et al., 2012; Hougland et al., 2012). Injury to one part of the spinal cord can have significant impact on systems that were not directly affected (Cote et al., 2012). The nervous system is particularly susceptible to such bystander effects because of the close physical proximity of neurons involved in diverse functions and the array of circuit interconnections, some of which may not be obvious until there is an injury. It is therefore beneficial to consider multiple elements of a system (such as examining sensory neurons and the spinal cord together when considering sensorimotor responses to SCI), as they can act differently in response to the same injury (Hougland et al., 2012).

The SCI condition involves pathologies beyond the spinal cord itself and the spinal cord disconnected from the brain can still generate output which relies heavily on the input it receives from the periphery. Understanding the status of the afferents providing input to the spinal cord and brainstem is of paramount importance. If the “below-level” spinal cord and the post-SCI vagal system are to be maintained in a healthy condition, then we must understand the vital roles that gateway primary afferent neurons play in both acute and chronic post-SCI pathologies in order to prevent sensory-based pathologies and direct these neurons to enhance recovery of function.
